# A critical exploration of the diets of UK disadvantaged communities to inform food systems transformation: a scoping review of qualitative literature using a social practice theory lens

**DOI:** 10.1186/s12889-023-16804-3

**Published:** 2023-10-11

**Authors:** Louise Hunt, Clare Pettinger, Carol Wagstaff

**Affiliations:** 1 School of Health Professions, Faculty of Health, University of Plymouth, Plymouth, UK; 2 Department of Food and Nutritional Sciences, University of Reading, Reading, UK

**Keywords:** Disadvantaged communities, Qualitative evidence, Scoping review, Food system research, Social practice theory

## Abstract

The UK food system affects social, economic and natural environments and features escalating risk of food insecurity. Yet it should provide access to safe, nutritious, affordable food for all citizens. Disadvantaged UK communities [individuals and families at risk of food and housing insecurity, often culturally diverse] have often been conceptualised in terms of individual behaviour which may lead to findings and conclusions based on the need for individual change. Such communities face public health challenges and are often treated as powerless recipients of dietary and health initiatives or as ‘choiceless’ consumers within food supply chains. As transforming the UK food system has become a national priority, it is important a diverse range of evidence is used to support understanding of the diets of disadvantaged communities to inform food systems transformation research.

A scoping review of UK peer reviewed qualitative literature published in MEDLINE, CINAHL Plus with Full Text, EMBASE, PsycINFO and Web of Science between January 2010 and May 2021 in English. Eligibility criteria were applied, a data extraction table summarised data from included studies, and synthesis using social practice theory was undertaken.

Forty-five qualitative studies were reviewed, which included the views of 2,434 community members aged between 5 and 83. Studies used different measures to define disadvantage. Synthesis using social practice theory identified themes of food and dietary practices shaped by interactions between ‘material factors’ (e.g. transport, housing and money), ‘meanings’ (e.g. autonomy and independence), and ‘competencies’ (e.g. strategies to maximise food intake). These concepts are analysed and critiqued in the context of the wider literature to inform food systems transformation research.

This review suggests to date, qualitative research into diets of UK disadvantaged communities provides diverse findings that mainly conceptualise disadvantage at an individual level. Whilst several studies provide excellent characterisations of individual experience, links to ‘macro’ processes such as supply chains are largely missing. Recommendations are made for future research to embrace transdisciplinary perspectives and utilise new tools (e.g., creative methods and good practice guides), and theories (e.g., assemblage) to better facilitate food systems transformation for disadvantaged communities.

**Supplementary Information:**

The online version contains supplementary material available at 10.1186/s12889-023-16804-3.

## Background

The food system includes *‘all the elements (environment, people, inputs, processes, infrastructures, institutions, etc.) and activities that relate to the production, processing, distribution, preparation and consumption of food, and the outputs of these activities, including socio-economic and environmental outcomes’* ([1], p11). This system affects UK social, economic and natural environments [2], and aims to provide access to safe, nutritious, affordable food for all citizens [3, 4]. Conceptualisations of this system show potential links between citizens’ diets and wider ‘macro’ elements such as supply chains [5]. Socially and economically disadvantaged communities are often treated as powerless recipients of dietary and health initiatives or as ‘choiceless’ consumers within food supply chains. Indeed, they are failed by the system because it is distorted by inequalities in access, demonstrated by escalating risk of food insecurity [6] and the inability to afford healthier foods [7]. Dietary patterns are associated with sociodemographic characteristics [8] with lower sociodemographic groups less likely to consume diets aligned with public health guidance [9]. Indeed, 15.5% of Western European deaths have been attributed to poor dietary habits alone [10].

Transforming the food system is of current strategic relevance in the UK [11], with numerous publications on this topic since 2019, for example [12] and [13]. Haerlin [14], highlights the scope and complexity of this task calling for a paradigm shift integrating the *‘previously segregated sectors of production, processing, trade, consumption, environmental assessment and health, as well as knowledge systems’* ([14], p18) as well as engaging with the communities the system serves [15]. The health impacts of the current food system on disadvantaged communities warrants examination to improve ecological public health nutrition strategies. Quantitative datasets using dietary survey methodologies are the predominant source of information about UK adult diets [16] yet may mis-represent diets in disadvantaged communities because sub-sample sizes are small [17] and fail to consider wider structural perspectives [18].

The necessity and complementarity of qualitative research to contextualise quantitative evidence is well known [19]. Recent qualitative reviews of diets in disadvantaged communities have explored individual perspectives such as healthy eating beliefs and food meanings [20], parents’ perceptions of the food environment and their influence on food decisions [21], and the healthy eating strategies employed through dietary change interventions [22]. Each review has a specific focus, supporting the pertinence of a broader approach using scoping review methods [23].

Within food studies, Neuman [24] advocates engagement with social theory, and social practice theory (SPT) in particular which offers potential to deepen understanding and facilitate social change. Attempting to explain society and culture in the context of structure and individual agency, theories of practice focus on practices as carried out by the people performing them [25]. Specifically, SPT purports three elements make up social practices; ‘materials’ (the stuff objects are made of, physical entities and technologies), ‘competencies’ (techniques, skills and know-how) and ‘meanings’ (ideas, aspirations and symbolic meanings) and that the material element of social life should be taken seriously as practices emerge, shift and disappear [26].

Traditionally, public health nutrition has focussed on improving individuals’ diet and food consumption rather than on the food system or environmental context in which food exists [27]. Yet the limitations of behaviourist conceptualisations of social change in relation to diet are long recognised [28], and the extent to which daily life, including eating, is embedded in the changing shapes of social practice has not always been acknowledged [24]. The breadth and complexity of the challenge of food system transformation [14] combined with SPT’s promise as an approach for the basis of social change [29], prompted the use of SPT to inform this synthesis and, in keeping with the review’s aim, better understand qualitative evidence of the diets of disadvantaged communities.

## Aim

To use scoping review methods to review UK qualitative literature examining the diets of disadvantaged communities using a social practice theory lens to inform food systems transformation research. To enable answering the research question: What qualitative data exists to explore the diets of disadvantaged communities? Specifically, to consider how the literature categorises and conceptualises disadvantage, and to what extent it acknowledges and links individual experience and practice to broader macro processes and issues such as supply chains and food system sustainability aspects (for example food processing). The review was carried out as part of preliminary benchmarking activities for a national United Kingdom Research and Innovation Strategic Priorities funded consortium food system transformation project [30].

## Methods

Scoping reviews are recommended for identifying and mapping relevant types of evidence, and the way research has been conducted [23]. To ensure best practice, the Preferred Reporting Items for Systematic Reviews and Meta-Analyses—Extension for Scoping Reviews (PRISMA-ScR) checklist and guidelines outlined by Tricco et al [31] was employed alongside the six-stage framework developed by Arskey & O’Malley [32] expanded upon by Levac, Colquhoun & O’Brien [33]. The framework involves identifying the research question, searching for relevant studies, selecting studies, charting data, and collating, summarising and reporting outcomes [33]. Stakeholder collaboration to refine and validate outcomes and facilitate two-way knowledge transfer, forms the final stage of the framework [33].

The review protocol was agreed by the research team (available from authors on request). Inclusion criteria stipulated studies must focus on the diets of people of any age living in UK disadvantaged communities. For the purposes of this review, diets were defined as ‘the food and drink usually eaten or drunk by a person or group’ [34], and disadvantaged communities as ‘individuals and families at risk of food and housing insecurity, often culturally diverse, who can experience multiple challenges; financial, mental health, physical health’ [30]. Studies were included that sought to work in disadvantaged communities defined by any measure, be about food, diet and/or the food environment, be qualitative or mixed methods with a significant qualitative element [for example interviews, case studies, observations, ethnography – see Table 1], be written in English and published in 2010 or later. Studies were excluded if they were carried out in institutional settings (e.g., schools, hospitals, prisons), did not take place in disadvantaged UK communities, were not specifically about diet, food or focussed on special diets (including weight management), or were quantitative studies. Studies focussing on policy alone were also excluded.

**Table 1:** Characteristics of included studies (*n=*45)

Group; exploring….	Author (year)	Aim	Study Design and method	Participants	How disadvantage measured	Individual, community or organisational focus
**Food insecurity**	Douglas et al., (2020) [35]	Explore challenges of food insecure people re health conditions.	Qualitative study. Grounded Theory. Interviews.	Food insecure adults with health conditions (*n=*20)	Participants in receipt of food aid	Individual
	Douglas et al., (2018) [36]	Capture perspectives of third sector workers delivering food insecurity services	Qualitative part of mixed methods study of food insecurity. Interviews.	Third sector workers (*n=*25)	Participants worked with people in receipt of food aid.	Individual, some community.
	Garthwaite et al., (2015) [37]	Explore relationship between ill health and food insecurity	Ethnographic part of larger health inequalities study. Observations, interviews	Adults using food bank, foodbank volunteers (*n=*42 users, 11 volunteers)	Participants in receipt of food aid. Area IMD.	Individual
	Harvey (2016) [38]	Understand families' experiences of food insecurity.	Mixed methods. Interviews.	Food insecure families (*n=*72 parents, 19 children)	Participants in receipt of food aid/previously identified as food insecure. Area with high child poverty rates.	Individual
	Jolly (2017) [39]	Understand experiences of food poverty for families at risk of destitution.	Practitioner research model. Part of superdiversity study. Interviews.	Parents from families at risk of destitution (*n=*7)	Participants refugees at risk of destitution.	Individual
	Knight, O'Connell and Brannen (2018) [40]	Explore experience of food poverty.	Qualitative case study approach. Interviews.	Young people and parents (*n=*45)	Area child poverty level.	Individual, some community.
	Laverty (2019) [41]	Explore informal ways young people manage food insecurity.	Qualitative ethnographic study. Observations, focus groups.	Young people (n not stated)	Area IMD	Community
	Blake (2019) [42]	Consider relationship between poverty and food insecurity and impact on community self-organisation.	Qualitative case study. Interviews and observations.	Community members and community organisation representatives (*n=* at least 41, full number not given)	Area IMD	Community
	Morares et al., (2021) [43]	Explore lived experience of food insecurity.	Interpretive qualitative research. Part of larger study. Interviews.	Adults experiencing food insecurity (*n=*24)	Participants in receipt of food aid, high deprivation area (IMD).	Individual
	Power et al., (2018) [44]	Understand lived experience of food in context of poverty.	Qualitative study. Focus groups, interview.	Pakistani and White British women (*n=*16)	IMD and health indicators of area plus participants’ house hold work situation.	Individual
	Power et al., (2020) [45]	Consider food insecurity.	Qualitative phenomenological study. Focus groups, interviews.	Food insecurity service providers, Pakistani and White British women at risk of food insecurity (*n=*16 women, 9 service providers)	Area IMD	Individual
	Puddlephatt et al., (2020) [46]	Explore factors influencing food choice in food insecure population.	Qualitative study. Interviews.	Food bank clients (*n=*24)	Participants in receipt of food aid.	Individual
	Purdam, Esmail and Garratt (2019) [47]	Explore food insecurity for UK older people (over 50), and experiences of using food banks.	Mixed methods. Case study approach. Interviews.	Food bank users aged 50 -75 years (*n=*36 service users, 6 service volunteers)	Participants in receipt of food aid.	Individual
	Pybus et al., (2021) [48]	Explore experiences of food and poverty to build community capacity.	Mixed methods. Co-design and participatory methods. Focus groups, survey.	Adults self-identifying as low income (*n=*22 for focus groups, 612 for survey)	Participants had low-income	Individual, some community
	Thompson et al., (2018b) [49]	Explore health and wellbeing challenges of food poverty and professional and organisational response.	Qualitative ethnographic part of wider study. Observations, interviews.	Food bank volunteers and users (*n=*14 families using food bank, 8 volunteers). Health and social care professionals (*n=*22)	Participants in receipt of food aid/ running food banks.	Individual
**Emergency food aid**	Douglas et al., (2015) [50]	Provide insight into experience of foodbank use.	Qualitative study. Grounded Theory and ethnographic approaches. Observations and interviews.	Adults using a food bank (*n=*7)	Participants in receipt of food aid.	Individual.
	Oncini (2021) [51]	Illuminate how food aid organisations responded to COVID - 19 crisis.	Mixed methods. Interviews.	Third sector emergency food provider directors or spokespeople (*n=*55)	All food banks surveyed	Organisational
	Purdam et al., (2016) [52]	Examine food aid discourse, demonisation of poverty and experiences of food bank users.	Qualitative ethnography, case study approach. Observations, interviews.	Food bank users (*n=*34)	Participants in receipt of food aid.	Individual and organisational.
	Wainwright et al., (2018) [53]	Explore food bank use.	Qualitative study. Interviews.	Food bank users (*n=*25)	Participants in receipt of food aid.	Individual.
**Local food environment**	Thompson et al., (2018a) [54]	Describe framing of chicken shops, characterise their integration into health.	Qualitative part of wider study. Go-along interviews, focus groups	Parents, children aged 11-15 (*n=*106)	‘low income’ neighbourhood	Individual and community
	Townshend (2017) [55]	Understand community impact of clustering of unhealthy shops.	Mixed methods, case study approach. Observations, interviews.	Young people, local politicians (*n=*4 local politicians, 10 community members)	Deprived areas of city	Community
	Estrade et al., (2014) [56]	Barriers independent fast food vendors may face when making menus healthier.	Qualitative study of fast food vendors near secondary schools. Interviews.	Fast food managers (*n=*10)	IMD and proportion of free school meals in area.	Community
**Healthy diet**	Cross-Bardell et al. (2015) [57]	Experiences of strategies for enhancing physical activity and diet.	Qualitative study. Interviews.	Community members of South Asian origin, health professionals (*n=*34 people of South Asian heritage, 11 health professionals)	Area IMD.	Individual
	Davison (2015) [58]	Understand determinants of food choice and dietary health promotion needs of young people not in education, employment or training.	Explorative qualitative study. Focus groups and interviews	Service providers, young people (*n=*14 young people, 7 service providers)	Attending Pupil Referral Unit (educational deprivation).	Individual
	Barton et al. (2011) [59]	Explore food choice and barriers to healthful eating.	Qualitative study, Grounded Theory analysis. Focus groups.	Community members (*n=*42)	Area IMD	Individual and community
	Dolan (2014) [60]	Explore men's experiences regarding gender differences and health	Qualitative study, comparative methods with focus on context. Interviews.	Working class men (*n=*22)	Mix of socioeconomic measures used to identify working class areas.	Individual, some community.
	Goldthorpe et al., (2018) [61]	Explore parent's experiences of providing healthy diet for pre-school child.	Qualitative study informed by psychological theories. Interviews.	Parents of pre-school children (*n=*21)	Area IMD	Individual.
	Grace (2011) [62]	Explore factors influencing lifestyle choice.	Qualitative study. Focus groups, interviews.	Adult members of Bangladeshi community without diabetes (*n=*70), religious leaders (*n=*29), health professionals (*n=*8).	Deprived part of London	Individual.
	Grant et al., (2018) [63]	Engage with subjective experience of health in pregnancy.	Interpretivist qualitative study. Creative methods, interview.	Pregnant women (*n=*10)	Participants’ IMD (most deprived quintile)	Individual
	Hardcastle and Blake (2016) [64]	Explore perceptions underlying food choices.	Qualitative part of larger study, inductive analysis. Interviews.	Parents of child participants of cookery programme (*n=*16)	Area IMD.	Individual
	Kahoum et al., (2015) [65]	Explore barriers to dietary choice for parents. Inform intervention development to promote healthier food environment for children.	Qualitative study, inductive analysis. Interviews.	Parents of infants and pregnant mothers (*n=*96)	Townsend deprivation index.	Individual, some community.
**Eating and foodways**^a^	van Kesteren and Adams (2020) [66]	Use practice theory to explore non-cognitive factors of everyday cooking performances, examine how these may affect healthy eating inequalities.	Mixed methods. Ethnography. Observations, interviews.	Mothers (*n=*31 via observation and interviews, 310 via survey)	Area IMD.	Individual.
	Sprake et al., (2014) [67]	Insight into nutrient intakes of homeless people, understanding of factors affecting food choice, determine contribution of charitable meal services to overall diet.	Mixed methods. Interviews.	Homeless people accessing charitable meal service (*n=*12)	Being homeless and receiving charitable meals.	Individual.
	Wills et al., (2011) [68]	Explain food and eating practices of families with young teenagers.	Qualitative study. Interviews	Teenagers, their parents or grandparents (*n=*36 teenagers, 35 parents or grandparents)	Area defined by % of children having free school meals. Individual participants; range of socio-economic variables collected.	Individual
	Lofink (2012) [69]	Examine how micro-environments influence diets.	Qualitative ethnography. Observations, interviews.	British Bangladeshi young people (*n=*165)	Economically depressed area of London.	Individual and community.
	Lovelace and Rabiee-Khan (2015) [70]	Explore food choices and understand socioeconomic and environmental influences constraining families.	Qualitative, Grounded Theory approach. Interviews	Mothers of pre-school children (*n=*11)	Participants had low income (not home owners, receiving income support/healthy start)	Individual and community
	Clement et al. (2014) [71]	Explore alcohol use in educationally marginalised population	Qualitative study, Grounded Theory analysis. Focus groups, interviews.	Young people, staff members (*n=*13 young people, 7 staff)	Participants educationally deprived.	Individual
**Infant feeding**	Cook et al. (2021a) [72]	Explore breastfeeding experiences and access barriers to local breastfeeding support services	Qualitative part of larger study. Focus groups.	Mothers (*n=*63)	Area IMD	Individual
	Cook et al. (2021b) [73]	Examine parents' complementary feeding knowledge beliefs and practices.	Qualitative part of larger study. Focus groups.	Parents (*n=*110)	Area IMD	Individual
	Hufton and Raven (2014) [74]	Understand refugee mothers’ infant feeding issues and experiences of their health professionals	Qualitative study. Interviews, focus groups.	Refugee mothers (*n=*30), health professionals (*n=*5)	Refugee status.	Individual.
**Food shopping and choice**	Thompson et al., (2013) [75]	Explore how residents of deprived neighbourhood shop for food, how the supermarket environment influences choices.	Qualitative ethnographic part of wider study. Go-along interviews.	Adult neighbourhood residents (*n=*26)	Area IMD, 30% of population on benefits.	Individual
**Emergency meal provision**	Pelham-Burn et al., (2014) [76]	Understand factors affecting composition of charitable meals, determine likely acceptability of possible improvements.	Mixed methods. Interviews.	Kitchen staff at day centre for homeless people (*n=*2 for interviews)	Study of emergency food provider.	Organisational
**Food’s environmental impact**	MacDairmid et al., (2016) [77]	Explore awareness of foods’ environmental impact.	Qualitative study. Focus groups, interviews.	Adults (*n=*83)	Area IMD	Individual.
**Impact of welfare change**	Moffat et al., (2016) [78]	Explore impact of bedroom tax.	Qualitative study, interpretive approach. Interviews, focus groups.	Adult social housing tenants and social housing providers (*n=*36 tenants, 12 providers)	Area IMD	Individual, some community
**Food involvement.**	Jarman et al., (2012) [79]	Explore women with lower educational attainment’s food involvement.	Qualitative study. Focus groups.	Women with young children with low educational attainment (*n=*28)	Participants educationally deprived.	Individual.

^a^Foodways are the culinary practices and eating habits of a people, region or historical period [80]

A search strategy was developed in consultation with an information specialist. Search terms were formulated by testing them across databases, and term truncations adapted for different databases (see Additional file 1 for an example search). In May 2021, five electronic databases were searched: MEDLINE, CINAHL Plus with Full Text, EMBASE, PsycINFO and Web of Science. After removal of duplicate records, and in keeping with the iterative approach outlined by Levac et al [33], the review team decided to focus only on peer reviewed literature and to exclude conference abstracts, opinion pieces, editorials and grey literature.

For each publication, a descriptive form was completed with the following items: title, type of publication, journal name, author, year of publication, methods, participants, geographical areas, theoretical positions and whether the paper focused mainly on organisations, communities or individuals. Key findings were then extracted and summarised within the form. In addition, particular attention was paid to how disadvantage was conceptualised and whether links with macro processes such as supply chains and food system sustainability aspects (for example food processing) were apparent. Validation was carried out on 10% of papers whereby data were extracted independently by CP and LSH, discussed, and agreement reached. LSH extracted data from the remaining papers.

Initial thematic analysis was undertaken using an inductive approach. The six steps identified by Braun and Clarke [81] were employed. This included coding the findings sections of all included papers (for mixed methods papers, only qualitative findings were coded). Following this, the three elements of social practice theory were used to structure emerging codes and themes [26] and, in accordance to aims, additional ‘macro’ elements noted. Coding attended to ideas arising from the texts as well as SPT elements. LSH led the analysis, with interpretations discussed with CP.

Initial thematic outcomes were presented to purposively selected *n=*5 community stakeholders during two online workshops (January 2021). The stakeholders were long-serving experienced front line practitioners running and delivering third sector food support, including soup kitchen, emergency food aid and broader food aid services. These workshops involved ‘sense checking’ our preliminary themes, focussing on how context impacts diets with feedback informing subsequent synthesis. Furthermore, outcomes were discussed with an independent researcher, who supported our use of the SPT lens. Outcomes were discussed in terms of relevance and relation to experiences, permitting refinement and validation [33].

## Findings

**Fig. 1:**
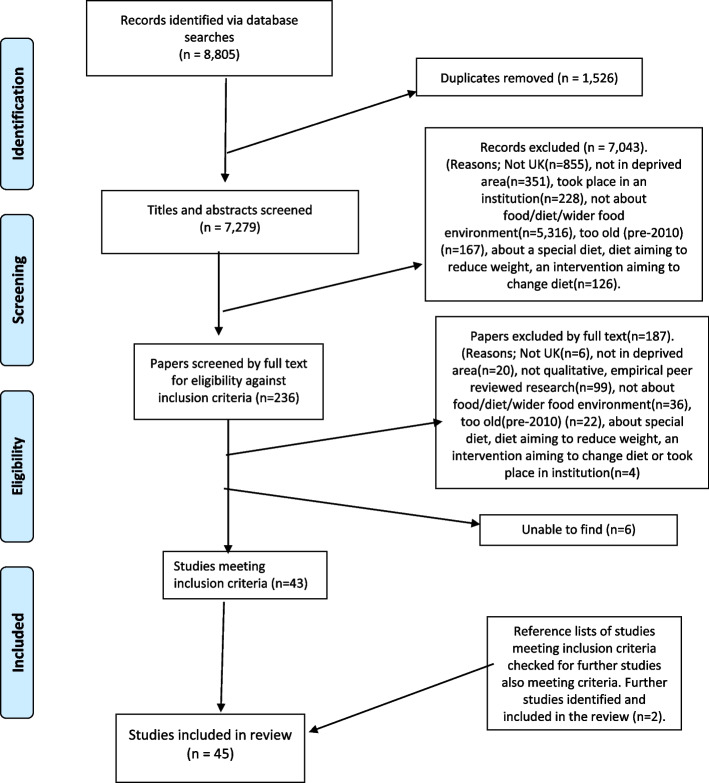
Prisma diagram

Records were retrieved and screened from 8,805 sources. Once duplicates were removed and inclusion criteria applied, 8,760 were excluded, resulting in 45 included peer reviewed studies representing the views of 2,434 participants from disadvantaged communities (see Fig. 1).

The age of community participants ranged from 5 to 83 years. Twenty-eight studies focused on individuals, four on communities, ten to some degree on both (see Table 1). Two studies had an organisational focus (e.g., food banks), and one individual and organisational. Six studies to some extent linked experience to ‘macro’ processes such as supply chains (see Tables 1 and 2). All studies employed to some degree traditional interview and focus group methods, with forty analysing findings thematically.

**Table 2:** Table mapping the codes, sub themes and overarching SPT headings making up the synthesis

*Codes*	*Sub themes*	*SPT Heading*
Costly public transport [40, 46, 48].	**Lacking affordable transport**	**Materials**
Lack of transport inhibiting supermarket access [40, 45, 46, 48, 65].		
Lack of transport inhibiting food bank access [45, 46, 49, 50, 52].		
Insecure housing [38, 39, 49, 50, 67].	**Being limited by housing**	**Materials**
Unaffordable housing [47, 48, 50, 78].		
Facilities limiting food [38, 39, 42, 47, 49, 50, 66, 67, 76].		
Low incomes putting pressure on diets [35–37, 39–44, 46, 47, 49, 50, 52, 65, 67, 74, 78].	**Low and or unpredictable income driving compromised diets**	**Materials**
Impact of low wages and employment practices [36, 43, 44, 48, 49, 52, 65, 78].		
Negative impact of low benefits payments [36, 37, 40, 48, 53, 67, 78].		
Benefit delays causing hardship [36, 40, 46–48, 50, 52, 53].		
Negative impact of welfare reforms [36, 37, 45, 46, 48–50, 52, 53, 74, 78].		
Variable access to shops and supermarkets [39, 46, 65, 69].	**Having variable access to local shops**	**Materials**
Local shops meeting needs [40, 55, 59, 69].		
Limited local shops negatively affect diet [62, 65].		
Local shops too expensive [40, 42, 45, 46, 48].		
Difficulty accessing supermarkets [39, 42, 45, 46, 48, 62, 65].		
Adequate supermarket access [37, 40, 46, 59, 70].		
Supermarket access enabling budget maximising strategies [37, 40, 42, 46, 48, 50, 52, 75].		
Downside to supermarket shopping [37, 75].		
Having abundant, accessible takeaways [40–42, 54, 55, 58, 65, 69].		
Takeaway abundance driving use when alternatives limited [65].		
Food competing with bills [35, 40, 43, 46–48, 50, 52, 78].	**Family or household feeding practices - The importance of making sure everyone is fed.**	**Meaning**
Prioritising children [35, 36, 39, 40, 44, 46–49, 52, 65, 74, 78, 79].		
Valuing filling carbohydrates [35, 37, 39, 45, 46, 52, 76].		
Eating low-cost convenience and processed foods [35, 37, 39, 42, 46, 59, 64, 65, 67, 70].		
Children’s’ ready acceptance of convenience and processed foods [40, 61, 68, 69].		
Ready acceptance drives use when too risky to chance waste [46].		
Children’s limited food experiences [39, 42, 46, 64].		
Shame and stigma of food bank use [35–37, 39, 43–48, 50, 52, 53, 78].	**Food in relation to autonomy, independence and community.**	**Meaning**
Recipients having no choice over food bank food [35–37, 39, 43, 46, 49, 50, 67].		
Food bank providing inappropriate foods [39, 46, 49, 50].		
Getting food from family [36, 40–47, 70, 78].		
No family or family unable to help [36, 45–47, 52, 78].		
Possible negative consequences of family support [45, 47].		
A family culture continuum from individualistic to communal [36, 40, 42–47, 52, 78].		
The social and community value of takeaways [39, 41, 54–56].		
Wanting to be healthy [37, 39, 43, 46, 48, 50, 61, 64, 65, 67, 69, 70, 74, 75, 77].	**Healthiness and freshness of food.**	**Meaning**
Eating fruit and vegetables [35, 37, 39, 40, 43, 46, 48, 50, 52, 58, 59, 61, 62, 64–68, 70].		
Cost limiting fruit and veg consumption [35, 37, 37, 42, 46, 48, 50, 52, 58, 59, 62, 64, 66, 67, 70].		
Cost affecting form of fruit and veg [37, 59, 70].		
Fresh fruit and veg increases wastage risk [37].		
A background of poor mental and physical health [35, 37, 46, 49, 50].	**Having poor mental and physical health.**	**Competencies**
Cycle of poor health and poor diet [35, 37, 46, 47, 49].		
Negative health consequences of inappropriate food bank foods [35, 37, 46, 47, 49, 50, 52].		
Resourceful use of shopping strategies [35–37, 39, 40, 43–48, 50, 52, 75, 78].	**Using strategies to maximise intake while minimising expenditure.**	**Competencies**
Rationing food [35, 38, 40, 46, 47, 50, 52, 65, 78].		
Getting food through community organisations [35–53, 65, 67, 76].		
Community organisations’ cooking and gardening opportunities [36, 39, 42, 49, 65].	**Learning.**	**Competencies**
Learning from family and friends [39, 57, 59, 61–66, 69–74].		
Family learning not deterministic [65, 66].		
Experience of food processing off-putting [59].	**Wider (macro) influences.**	**Linking experience to broader macro processes**
Social value of meat trumping sustainability issues [77].		
Community organisations having little choice of food bank food provided [35, 36, 50, 76].		

### Conceptualisation of disadvantage

All studies took place in disadvantaged communities, which were defined in varying ways; some identified a geographic area of deprivation and recruited participants living within it. The most common measure used was the Index of Multiple Deprivation (IMD) (*n=*18), but other definitions included: high child poverty rates (*n=*2); high free school meal rates (*n=*2); Townsend deprivation index (*n=*1) and the percentage of people in the area claiming benefits (*n=*1). Some studies used vague terms to define area level disadvantage, such as ‘a range of official statistics’ to identify ‘working class areas’, or ‘low-income neighbourhoods’, (*n=*3). In other studies participants formed part of a deprived community because of their individual characteristics. For example, low-income levels (*n=*2), educational disadvantage (*n=*3), being previously identified as food insecure, being in receipt of emergency food aid or charity meal services (*n=*12), refugee or immigration status (i.e., being at risk of destitution) (*n=*2), or homelessness (*n=*1). In total eighteen studies defined deprivation using individual characteristics, twenty-three studies used area level characteristics, and four used both. When combined with information about whether a study focussed at an individual, community or organisational level (see above and Table 1), these definitions may provide some insight into conceptualisations of disadvantage.

### Thematic findings

**Fig. 2:**
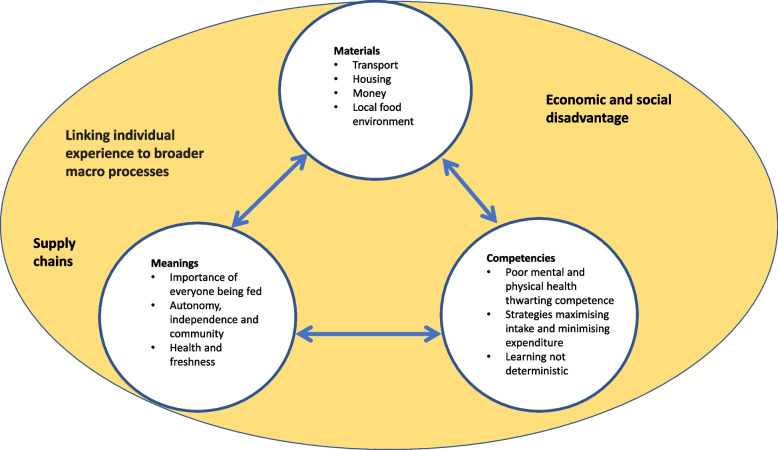
Diagram illustrating SPT headings and sub-themes

Synthesis using social practice theory identified often over-lapping themes of food and dietary practices shaped by interactions between ‘material factors’ (transport, housing and money); ‘meanings’ (e.g. autonomy and independence), and ‘competencies’ (e.g. strategies to maximise food intake). See Fig. 2 and Table 2. Each SPT heading is presently outlined with its key determining features, with consideration of links with ‘macro’ processes forming a fourth heading (to inform food system transformation research).

#### Materials

This heading addresses ‘material’ considerations within the reviewed papers, namely sub-themes of: transport, housing, money and the local food environment.

Lack of access to affordable public transport could inhibit access to supermarkets selling cheaper food and community organisations such as food banks. Lack of access to affordable, suitable housing could limit the facilities and equipment needed to prepare and store food. Money issues included low and unpredictable incomes, low benefits payments and delays or sanctions putting pressure on food affordability. Local food environments provided variable access to local shops and supermarkets, which could curb choice and reduce availability of healthy or fresh food. Sometimes local shops were present, but prohibitively expensive, supermarket access could facilitate buying the cheapest food and value-maximising strategies such as bulk buying, but persuasive marketing and poor-quality food offerings were also reported. Takeaway outlets were cited as abundant and easily accessible. Such accessibility could drive use when access to alternative food outlets was limited.

#### Meanings

This heading outlines three groups of ‘meanings’ (sub-themes) drawn from the papers; i) the importance of making sure everyone is fed, ii) autonomy, independence and community, and iii) health and freshness.

Firstly, meanings are associated with the importance of making sure everyone is fed. Food competed with other costs such as rent, lighting and heating, and in the context of tight budgets, parents, especially mothers, reportedly went without food to prioritise children. Participants valued carbohydrate foods such as bread because they were filling and low cost, and the low price of convenience, frozen, and processed foods was noted by some studies. Children’s ready acceptance of these foods may drive consumption in families for whom food wastage could not be risked. Perhaps in consequence, some studies reported children’s limited food experiences.

Secondly, meanings covered autonomy, independence and community. The shame and stigma of accessing emergency food support was frequently reported. Studies also noted such support often resulted in lack of choice and limited access to culturally appropriate foods. Families and friends were reported to facilitate access to food when needed, yet studies acknowledged not everyone has family, or a family able to help. Obtaining food from family could result in feelings of dependency, and cultural differences were noted; some families reportedly expected independence of all adult members, while others provided extensive food and other support. An important social and community element of eating takeaway food was reported that may drive consumption as it may not be available elsewhere; with specific meanings in terms of community and belonging, takeaway outlets are places to meet friends, can be owned by friendly local people, provide local job opportunities, and constitute a way of supporting the local community.

Thirdly, meanings concerned health and freshness. When asked about health and diet, participants wanted to be healthy, and discussion of fruit and vegetable intake was dominant. However, cost impacted the volume and or frequency of consumption, meaning people were not eating as much as they would like. Cost also influenced the form of vegetables eaten, because while fresh might be preferred, tinned and frozen options were cheaper and avoided risk of wastage.

#### Competencies

This heading draws from the papers how competencies may be impacted by poor mental and physical health. It highlights competence in strategies to maximise intake while minimising expenditure, suggests learning can happen, and that competencies are not deterministic.

Several studies reported significant levels of poor mental and physical health which could constrain shopping and cooking competencies, and a cycle whereby poor health can lead to food insecurity and food insecurity can negatively impact health. Accounts illustrated participants’ competence in eating a suitable diet could be thwarted when accessing food banks, because the foods available may not meet health needs. Competence in strategies to minimise expenditure and maximise intake was noted, such as shopping for best prices, budgeting, and accessing help from community organisations.

Overall, studies evidenced that competencies are not fixed; some community organisations offered learning opportunities via gardening and cooking projects, and although the family was noted as somewhere where dietary habits form and skills develop, learning about food within families was important, but not deterministic.

#### Linking individual experience to broader ‘macro’ processes

This additional heading explains links between individual experience and the broader ‘macro’ processes of food processing and supply chains in the context of economic and social disadvantage.

One study reported that direct experience of working in meat processing, for example, injecting raw chicken with water, can mean people value unprocessed food more highly, and sought out unprocessed meat to eat. Another highlighted the social and cultural role of meat, reporting reluctance to reduce consumption in light of sustainability concerns. Supply chains serving community organisations afforded them little choice over the emergency food they were able to provide.

## Discussion

This scoping review has explored UK qualitative literature of the diets of disadvantaged communities using a social practice theory lens to inform food systems transformation research. Specifically, it has considered how disadvantage has been conceptualised, and to what extent links between individual experience and broader macro processes have been acknowledged. Analysis using social practice theory resulted in headings corresponding to ‘materials’, ‘meanings’ and ‘competencies’. Consideration of links with ‘macro’ processes formed a fourth heading. There is overlap between sub-themes, with diversity in method and conceptualisation making synthesis and collation challenging. Our sub-themes, in part, illustrate social issues (e.g. access and affordability) that are already well evidenced. Yet, some aspects warrant deeper critique. Here, we briefly consider the conceptualisation of disadvantage in the context of ‘systems thinking’ for public health practice. Subsequently, we appraise each heading in turn, highlighting the overlaps. Thereafter, recommendations are made for research and practice.

The characterisation and conceptualisation of disadvantage across the studies was diverse with most of our reviewed studies focussing at the individual level and some at community level. There are well evidenced drawbacks on measures of deprivation such as the commonly used IMD e.g. [82]. Similarly, proxy measures as indicators of poverty, such as food bank use, child poverty rates, are known to be limited in scope [83].

Our findings suggest the gaze of qualitative researchers on disadvantaged communities maybe somewhat individualised. Indeed, several included studies provided excellent characterisations of individual experience (i.e., [37, 46]), and while many clearly linked their findings to structural issues and the need for structural change (for example, [37, 43, 45, 48]), it has been recognised that ideas about the social origins of inequalities (i.e., their political and structural causes) consistently struggle to compete with dominant behavioural perspectives in public health [84]. In public health terms, such focus on individuals has been critiqued in relation to the complex systems within which they are located [85]. The relational interactions of multiple levels within systems confirms that the relationship between individual and population health is largely relative and dynamic [86]. Indeed, the mechanisms operating at the individual and social levels are known to be analytically separate as they make different epistemological assumptions [87], warranting further critique and recommending development of understanding of more diverse theoretical perspectives to reflect the complexity.

Applying SPT to this literature enabled findings to be summarised into a coherent narrative, but more importantly, formed a small step towards moving thinking beyond individuals towards populations [29] whilst maintaining a focus on social sustainability. It is known that this level of intervention requires a ‘systems thinking approach’ [88] and is motivated by growing recognition of complexity [89].

Within our ‘materials’ heading, the overarching theme of cost uncovered the use of strategies to make food supplies stretch to feed family members. The fact that food is an ‘elastic’ item within the household budget is supported in the literature [90–92]. This issue has become increasingly marked through the Covid-19 pandemic, with fuel prices forcing a choice between ‘heating or eating’ [93] and leading to increased household debts [94], currently exemplified within the cost of living crisis [95]. This overlaps with the ‘competency’ heading whereby strategies to minimise expenditure and maximise intake were noted, such as shopping for best prices, budgeting, and accessing help from community organisations. Yet the (neoliberal) stigma [96] of obtaining food from community organisations where there is limited choice of food and the unacceptability within some families of family food support, suggest accessing food outside the traditional market economy may be socially incongruous.

Similarly, access is a ‘materials’ theme drawn out as a strong public health issue, with poor access reported in relation to transport and housing. There are known links between transport poverty and social exclusion [97]. Forty one percent of UK households lack access to a car, compromising access to healthy food [98]. Similarly, lack of access to affordable, suitable housing was suggested to negatively affect diets by limiting the facilities and equipment needed to prepare and store food [99]. There is evidence to suggest that poor households with less equipment are at greater risk of food insecurity [99–101].

In terms of the food environment, access to take away outlets was easy and could drive use when other food outlets were limited [65]. The reported meanings as regards the social and community functions of take away outlets are interesting and overlooked in the literature. Blow et al [102] provide an account of the sociocultural influences relating to takeaway food consumption, including their contribution to bonding, relationship building and being part of a community. This supports the interactions between individuals and their food environment as a complex adaptive system [103] requiring multiple level considerations for research. Thompson et al [54] suggest alternative social meeting places may be unavailable in communities with high levels of deprivation. This fits with evidence demonstrating the disproportional impact of UK government austerity policies on poorer cities [104], and the experience of increased social isolation in such areas due to statutory service losses [105]. The practice of eating takeaway food combines materials (lack of alternative meeting places and food outlets) with the meaning of being part of a community. We recommend consideration of the nuances of socio-(political)economic interactions for future food system research.

The ‘meanings’ heading of our review highlights social practices whereby the positive meaning of freshness and health push the practice of eating fresh fruit and vegetables, but materials (money and access) constrain their consumption. Healthier diets are known to be more expensive and require greater proportional spend from household budgets [106]. Across all the themes cost influenced diet in several ways; low incomes affected the affordability of fruit and vegetables, a known ‘marker’ of a healthy diet [107], carbohydrate foods were valued for being cheap and filling, and price was noted as a possible driver of convenience and processed food consumption. As well as cost, poor access to healthier foods [108] is another ‘materials’ overlap. Ultra-processed foods are readily available, and consumption is known to be high particularly in lower socio-economic groups, which can influence health outcomes [109]. This evidence points to healthy eating as an unachievable goal within present social, economic and cultural systems [110], which might explain low adherence to ‘healthy eating’ guidance [111]. Consequently, telling families living in poverty that they should make healthier choices increases ‘victim blaming’ and ignores the conditions that prevent them doing so and is insulting and even futile [112].

The ‘competencies’ heading highlighted mental and physical health as important issues. Food insecurity is known to be a risk factor for compromised mental health [113]. Similarly, poor nutrition is known to be associated with physical and mental health issues [114]. Thus, we see a vicious cycle whereby poor health can lead to food insecurity and food insecurity can negatively impact health and wellbeing. Importantly, findings emphasise how the current food aid system can inhibit people’s utilisation of current competencies in looking after their own health. This affirms the need for change. Overall, our reviewed studies evidenced that competencies are not fixed; some community organisations offered learning opportunities via gardening and cooking projects, which are known to offer (mental health) benefits [115], and although the family was noted as somewhere where dietary habits form and skills develop, learning about food within families was important, but not deterministic.

Only six included studies linked experiences and practices to broader ‘macro’ issues such as supply chains and food system sustainability aspects (for example food processing). This review supports the view of the Independent Food Aid Network (IFAN) that the charitable food support supply chain provides a lack of choice to the community organisations it serves, and that the food it provides can be culturally and medically unsuitable [116]. This highlights the precarity of obtaining food from a ‘hybrid’ of commercial shops and the food aid supply chain [117] and the political and ethical debates presented by emergency food aid provision [118] and supports the need to move beyond this model. IFANs cash first approach [116] has emphasised the need for change, as does the Trussell Trust’s recent strategic plan [119] which focusses on community policy and public understanding. This exemplifies the urgent need to build more sustainable supply chains by adopting community resilience (as supported by Blake et al [42]) and points to a recommendation to embrace active food citizenship [120]. Yet our review suggests that the views of people living in disadvantaged communities have not been widely sought on these social issues and that the link has not yet been made (hence this review). One included paper [48] employed co-production methods which are topical and known to enhance research relevance [121], and another [63], creative methods which can foster ethical research [122] and positively influence mental wellbeing [123]. We suggest the need for wider adoption of such community centred collaborative co-production methods to support food system transformation.

To fully embrace this, transdisciplinary research practices are required that address real-world problems by bringing together diverse knowledge, experience, methods and models [124] including knowledge from non-academic actors [125]. Mitchell et al [126] stipulate that such practices can generate relevant stocks and flows of knowledge which are accessible for all actors, thus influencing the likelihood of lasting change. This is particularly pertinent for food systems research where a transdisciplinary approach is increasingly advocated [127] to take into account the importance of evaluating the ethical and political rigour of mixed methods [89] so that new tools and models can be used to better facilitate food systems transformation and effective ‘systems thinking’ knowledge mobilisation [128].

### Recommendations (research and practice)

We suggest the need for wider adoption of community centred collaborative co-production methods that utilise and explore novel/emerging tools and theories to better support and facilitate food systems transformation for disadvantaged communities, for example:

1. More consistent training for public health researchers and practitioners on ‘food systems’ is required and should include:
  - Better understanding of complexity i.e., relational interactions of multiple levels within dynamic social systems e.g., drawing on ‘complexity theory’ [129]. This should include critique of more diverse theoretical perspectives e.g., SPT (as used in this paper) or e.g., assemblage theory [130] to reflect complexity of food systems transformation discourse.
  - Deeper consideration of the nuances of socio-(political)economic interactions for research and practice inherent within disadvantaged communities.
  - The benefits and challenges of embracing transdisciplinary approaches within food systems research and practice (to include engagement of communities and other food system stakeholders).
2. Co-creation (with communities) of practical toolkit(s) to support researchers and practitioners to embrace more community-centric ‘co-production’ approaches. Tools might include good practice guides; food citizenship conceptual knowledge; tips for creative approaches.

Other general recommendations require that future food systems research must address affordability and access issues as well as exploring the barriers and aspirations of disadvantaged communities in comparison to those of public health practitioners and other food system stakeholders. In particular, specific dietary preferences (and the context driving them) are important considerations to inform future research design, by identifying interventions to improve food affordability and improve access to fruit and vegetables.

### Strengths and limitations

This scoping review adopts a novel approach by using social practice theory (SPT) as a lens to collate and synthesise the qualitative literature. SPT can help move thinking away from individual behaviour towards a wider viewpoint [26] and to our knowledge this is the first time it has been used in this way. The review has strengths relating to the validated framework used to systematically search the five databases and clearly map data inclusion, extraction and collation processes. The consultation with community stakeholders to ‘sense check’ our preliminary themes is also a strength. The studies reviewed (*n=*45) represented *n=*2,434 views from community members.

However, inherent limitations remain; our search strategy aimed to locate all qualitative studies exploring diets of disadvantaged UK communities, yet we recognise our search was not exhaustive and other bodies of literature may hold additional data. Given the complexity of the topic, some articles may have been missed and scoping review methodology does not include quality appraisal, which might have been useful as the conceptual density of some studies resulted in their greater contribution compared to others. This review is also limited because it focusses on pre-pandemic research warranting follow up [131]. Another limitation is that only UK based studies were included, although themes maybe relevant to other high-income countries. Parameters for the categorisation of studies into those focussed mainly on individuals, communities, or organisations could have been better defined. Finally, grey literature searches might also have strengthened the findings to support the published literature [132]. Despite these limitations, this scoping review provides highly relevant insights to support better understanding of the contextual factors influencing the diets of disadvantaged communities. This review has supported benchmarking activities and provides practical recommendations that can be used by researchers and practitioners engaged in food systems transformation research activities.

## Conclusion

This scoping review has explored UK qualitative literature of the diets of disadvantaged communities using a social practice theory lens to inform food systems transformation research. Findings have suggested that to date, qualitative research into the diets of UK disadvantaged communities provides diverse findings that mainly focus on disadvantage at an individual level. Whilst several studies provide excellent characterisations of individual experience, links to ‘macro’ processes such as supply chains and food system sustainability aspects are largely missing. Recommendations are made for future research to consider better understanding of complexity by developing more innovative transdisciplinary research practices that utilise new tools (e.g., creative methods and good practice guides), systems thinking and other theories (e.g., assemblage) to more effectively tackle food system challenges. Such research practices need to consider wider structural factors including the nuances of socio-(political)economic interactions and affordability and access issues. Finally, knowledge exchange and wider adoption of co-production methods are essential to support food system transformation and amplify community voices to build community resilience, resourcefulness and capital.

### Supplementary Information

**Additional file 1.** Example search: Medline search 26.5.21.

## Data Availability

The datasets used and/or analysed during the current study are available from the corresponding author on reasonable request.

## Abbreviations

SPT: Social Practice Theory
IMD: Index of Multiple Deprivation
PRISMA-ScR: Preferred Reporting Items for Systematic Reviews and Meta-Analyses—Extension for Scoping Reviews
