# Supplementary material for: A critical exploration of the diets of UK disadvantaged communities to inform food systems transformation: a scoping review of qualitative literature using a social practice theory lens
Source: BMC Public Health. 2023 Oct 11;23:1970. doi: 10.1186/s12889-023-16804-3 (PMC10568843; doi:10.1186/s12889-023-16804-3)
Supplement: Supplementary file 1 [file 12889_2023_16804_MOESM1_ESM.odt]

Example search: Medline search 26.5.21.

| 1. | (people or persons or men or women or adult).mp. [mp=title, abstract, original title, name of substance word, subject heading word, floating sub-heading word, keyword heading word, organism supplementary concept word, protocol supplementary concept word, rare disease supplementary concept word, unique identifier, synonyms] |
| --- | --- |
| 2. | (older adults or older or elderly or aged or aging or middle aged).mp. [mp=title, abstract, original title, name of substance word, subject heading word, floating sub-heading word, keyword heading word, organism supplementary concept word, protocol supplementary concept word, rare disease supplementary concept word, unique identifier, synonyms] |
| 3. | (adult children or adolescent or young adult).mp. [mp=title, abstract, original title, name of substance word, subject heading word, floating sub-heading word, keyword heading word, organism supplementary concept word, protocol supplementary concept word, rare disease supplementary concept word, unique identifier, synonyms] |
| 4. | (child or minors or infant).mp. [mp=title, abstract, original title, name of substance word, subject heading word, floating sub-heading word, keyword heading word, organism supplementary concept word, protocol supplementary concept word, rare disease supplementary concept word, unique identifier, synonyms] |
| 5. | (family or friends or single person or parent or grandparent).mp. [mp=title, abstract, original title, name of substance word, subject heading word, floating sub-heading word, keyword heading word, organism supplementary concept word, protocol supplementary concept word, rare disease supplementary concept word, unique identifier, synonyms] |
| 6. | 1 or 2 or 3 or 4 or 5 |
| 7. | (low income or low-income or poverty or poorest or poverty areas or social class or employment insecurity or precarious employment).mp. [mp=title, abstract, original title, name of substance word, subject heading word, floating sub-heading word, keyword heading word, organism supplementary concept word, protocol supplementary concept word, rare disease supplementary concept word, unique identifier, synonyms] |
| 8. | (Factor socioeconomic or inequalities social or deprivation or marginalized or disadvantaged or underprivileged or vulnerable or vulnerable populations).mp. [mp=title, abstract, original title, name of substance word, subject heading word, floating sub-heading word, keyword heading word, organism supplementary concept word, protocol supplementary concept word, rare disease supplementary concept word, unique identifier, synonyms] |
| 9. | (temporary housing or housing insecurity or educational disadvantage or educational level or cultural diversity).mp. [mp=title, abstract, original title, name of substance word, subject heading word, floating sub-heading word, keyword heading word, organism supplementary concept word, protocol supplementary concept word, rare disease supplementary concept word, unique identifier, synonyms] |
| 10. | 7 or 8 or 9 |
| 11. | (diet or diet, healthy or diet, western or diet, vegetarian or infant food or food or drink or beverage or sustenance or meal or snacks).mp. [mp=title, abstract, original title, name of substance word, subject heading word, floating sub-heading word, keyword heading word, organism supplementary concept word, protocol supplementary concept word, rare disease supplementary concept word, unique identifier, synonyms] |
| 12. | (nutritious or food quality or fresh or convenience or product or packet or cook or cooking or food organic or food genetically modified).mp. [mp=title, abstract, original title, name of substance word, subject heading word, floating sub-heading word, keyword heading word, organism supplementary concept word, protocol supplementary concept word, rare disease supplementary concept word, unique identifier, synonyms] |
| 13. | (home cook or family meal or restaurant or take away or eat out).mp. [mp=title, abstract, original title, name of substance word, subject heading word, floating sub-heading word, keyword heading word, organism supplementary concept word, protocol supplementary concept word, rare disease supplementary concept word, unique identifier, synonyms] |
| 14. | (Diet, food and nutrition).mp. [mp=title, abstract, original title, name of substance word, subject heading word, floating sub-heading word, keyword heading word, organism supplementary concept word, protocol supplementary concept word, rare disease supplementary concept word, unique identifier, synonyms] |
| 15. | (nutrients or nutritive value or nutritional status or micronutrients or vitamins or dietary fiber or carbohydrates or sugars or proteins or energy intake or food ingredients).mp. [mp=title, abstract, original title, name of substance word, subject heading word, floating sub-heading word, keyword heading word, organism supplementary concept word, protocol supplementary concept word, rare disease supplementary concept word, unique identifier, synonyms] |
| 16. | (eat or dine or consume).mp. [mp=title, abstract, original title, name of substance word, subject heading word, floating sub-heading word, keyword heading word, organism supplementary concept word, protocol supplementary concept word, rare disease supplementary concept word, unique identifier, synonyms] |
| 17. | (hunger or over nutrition or obesity).mp. [mp=title, abstract, original title, name of substance word, subject heading word, floating sub-heading word, keyword heading word, organism supplementary concept word, protocol supplementary concept word, rare disease supplementary concept word, unique identifier, synonyms] |
| 18. | (food assistance or food deprivation or food deserts or food insecurity or food security or food supply).mp. [mp=title, abstract, original title, name of substance word, subject heading word, floating sub-heading word, keyword heading word, organism supplementary concept word, protocol supplementary concept word, rare disease supplementary concept word, unique identifier, synonyms] |
| 19. | Food industry.mp. |
| 20. | Food preferences.mp. |
| 21. | (Health knowledge, attitudes, practice or attitude to health or food ways or food acquisition or food literacy or food value).mp. [mp=title, abstract, original title, name of substance word, subject heading word, floating sub-heading word, keyword heading word, organism supplementary concept word, protocol supplementary concept word, rare disease supplementary concept word, unique identifier, synonyms] |
| 22. | 11 or 12 or 13 or 14 or 15 or 16 or 17 or 18 or 19 or 20 or 21 |
| 23. | (UK or United Kingdom or Britain or England or Ireland or Northern Ireland or Scotland or Wales).mp. [mp=title, abstract, original title, name of substance word, subject heading word, floating sub-heading word, keyword heading word, organism supplementary concept word, protocol supplementary concept word, rare disease supplementary concept word, unique identifier, synonyms] |
| 24. | 6 and 10 and 22 and 23 |
| 25. | limit 24 to (english language and humans and yr="2010 - 2022") |
